# Development and evaluation of the “Toothbrushing Timer with Information on Toothbrushes” application: A prospective cohort pilot study

**DOI:** 10.1002/cre2.797

**Published:** 2023-10-23

**Authors:** Yoshino Kaneyasu, Hideo Shigeishi, Masaru Sugiyama, Kouji Ohta

**Affiliations:** ^1^ Department of Public Oral Health, Program of Oral Health Sciences, Graduate School of Biomedical and Health Sciences Hiroshima University Hiroshima Japan; ^2^ Department of Oral Health Sciences, Faculty of Health Care Sciences Takarazuka University of Medical and Health Care Takarazuka City Hyogo Japan

**Keywords:** applications, mobile health, oral health, toothbrush

## Abstract

**Objectives:**

For people, it is challenging to be conscious of the appropriate toothbrushing time to maintain good oral health in daily life. The aim of this study was to preliminarily examine the utility of an application (app) that combines a toothbrushing timer and information on toothbrushes.

**Materials and Methods:**

We developed the “Toothbrushing Timer with Information on Toothbrushes” app to help users ensure appropriate toothbrushing time and learn about the beneficial characteristics of toothbrushes. A total of 18 participants were registered for the study. At baseline (T0) and after 1 month (T1) of app usage, study participants answered a digital questionnaire that comprised three questions on oral health practice, self‐efficacy in oral hygiene, and quality of life related to oral health (Oral Health Impact Profile‐14 [OHIP‐14]).

**Results:**

Five participants were excluded from the analysis as they did not answer the digital questionnaire. Finally, 13 participants completed the survey with a follow‐up of 1 month. The 13 participants were grouped into health professionals (*n* = 8) and non‐health professionals (*n* = 5). The total scores for oral health practice and self‐efficacy related to oral hygiene increased after a month of app usage in health professional and non‐health professional groups. However, there were no significant differences between T0 and T1 in either group. The total score of OHIP‐14 was lower at T1 than at T0 in both groups. Therefore, participants showed better oral health practice, self‐efficacy in oral hygiene, and quality of life related to oral health at T1 compared with that at T0.

**Conclusions:**

Our app showed positive results for the users and is useful in maintaining and promoting oral health awareness and practice. However, our pilot study lacks sufficient power and did not yield significant differences. Therefore, high‐quality clinical trials with larger sample sizes are warranted for further improvement and evaluation.

## 1 INTRODUCTION

Toothbrushing is the most common and widespread method for maintaining oral health globally (Van Leeuwen et al., 2019; Stroski et al., 2011). Moreover, toothbrushing reduces gingivitis risk (Stroski et al., 2011) and prevents periodontal disease (Van der Weijden & Slot, 2015). Previous studies have reported that toothbrushing more than three times a day reduces the risk of diabetes (Chang, Lee, et al., 2020). In addition, oral hygiene care, such as toothbrushing, is related to a reduced risk of occurrence of atrial fibrillation and heart failure (Chang, Woo, et al., 2020).

Toothbrushing duration is an important factor for effective dental plaque removal (Hayasaki et al., 2014). However, people generally spend a short time brushing their teeth (Saxer et al., 1998). Professionals recommend 3 min to be a sufficient duration for brushing (Ganss et al., 2009; Hayasaki et al., 2014), and people should adhere to this time. In line with the previous recommendation, another research reported that 3 min of brushing time is sufficient for adults (Madléna et al., 2004). Furthermore, people should replace toothbrushes regularly because bristle wear out following long‐term use can damage the gingiva (Graetz et al., 2017). Therefore, people are generally looking for the next new toothbrush periodically, and numerous toothbrushes have been developed and sold to date. Hayasaki et al. (2014) reported that there are more than 450 types of toothbrushes available in Japan. Accordingly, it is difficult for people to find the appropriate toothbrush for themselves from this vast selection of distinctive features and construction motifs. In particular, people who want to use an appropriate toothbrush to suit their needs with an ability to prevent periodontitis or cavity may find it overwhelming to choose an appropriate one among the variety of toothbrushes available. Thus, we considered that an application (app) that could facilitate easy measurement of toothbrushing time for 3 min without setting a timer and learn about the appropriate toothbrush for a particular use‐case would be beneficial.

To the best of our knowledge, to date, there have been few reports concerning apps that are specifically designed for the recommended toothbrushing time and facilitate the search for an appropriate toothbrush. Nevertheless, there are some apps that assist in how to brush teeth appropriately (Hotwani et al., 2020; Jacobson et al., 2019), an educational app for processing dental treatments (Takenouchi et al., 2020), and apps for monitoring gingival tissue to promote gingival health (Tobias et al., 2021; Tobias & Spanier, 2020).

The “Toothbrushing Timer with Information on Toothbrushes” app that we developed can easily measure toothbrushing time for 3 min and provide beneficial information on toothbrushes to the user, regardless of background information concerning the toothbrush and independent of the manufacturer or distributor of each toothbrush type. “Toothbrushing Timer with Information on Toothbrushes” app may motivate the user to ensure sufficient daily brushing practice. Thus, the purpose of this study was to develop an app combining a toothbrushing timer and information on toothbrushes and demonstrate its impact on oral health awareness and practice.

## 2 METHODS

### 2.1 Participants

This was a prospective pilot study, and the participants were recruited among the students and staff members of Hiroshima University from February 2022 to March 2023. We included participants who were healthy, 20 years or older and had at least 18 natural teeth. We excluded participants with serious systemic diseases. The participants were provided with written informed consent forms to sign, and the follow‐up was performed for 1 month. Written informed consent for participation was obtained from 18 individuals. The study protocol was approved by the Ethics Committee of Hiroshima University in February 2022 (No. E‐2766). The standards of the Strengthening the Reporting of Observational Studies in Epidemiology statement were used as the study checklist (von Elm et al., 2008).

The digital questionnaire in the present study was prepared in Microsoft Forms (Microsoft Inc.) and participants answered the questionnaire using a QR code at baseline (T0), before downloading our app. After connecting to the app through a URL sent by the researcher, participants installed the app on their own digital devices, such as a smartphone, tablet, or personal computer (PC), whichever they considered easier to use. Subsequently, participants were allowed to use our app for 1 month and, then, completed the questionnaire again using the QR code.

We expected that oral health awareness and practice would be more effectively improved in health professionals than in non‐health professionals by using our app, because health professionals may have a stronger interest in oral health. To consider the impact of the difference of occupation on the results of oral health awareness and practice, participants were divided into two groups. The first group included health professionals (e.g., those with national medical, pharmaceutical, or dental licenses) and medical, pharmaceutical, or dental students (who attend hospital training to obtain these national licenses), and the second group included non‐health professionals at Hiroshima University. All collected responses were anonymous and used to analyze changes between T0 and T1, as well as differences between the health professional and non‐health professional groups.

The determined sample size in this study was 20 participants, according to Humm et al. (2020), because their pilot study report concerning a toothbrushing application was also similar to our study protocol.

### 2.2 Questionnaire in oral hygiene: Oral health practice and self‐efficacy related to oral hygiene

The questionnaire for the present study was designed according to Haque et al. (2016) and Wei et al. (2021) to estimate the oral health practice and self‐efficacy related to oral hygiene. It contained the following eight items divided into two main categories: *Oral health practice*: (1) Frequency of toothbrushing (times per day) (maximum score: 3, minimum score: 1; once: 1, twice: 2, three or more times: 3). (2) Toothbrushing time (min) (maximum score: 3, minimum score: 1; 1 min: 1, 2 min: 2, ≥3 min: 3). (3) Using a cleaning aid such as dental floss or interdental brushes (maximum score: 1, minimum score: 0; yes: 1, no: 0). (4) Using an oral rinse (maximum score: 1, minimum score: 0; yes: 1, no: 0). (5) Frequency of replacing the toothbrush (maximum score: 3, minimum score: 1; 6 months or more: 1, more than 3 months and less than 6 months or when the bristles were splayed: 2, 1 month: 3). *Self‐efficacy related to oral hygiene*: (6) Do you think you keep your teeth clean by toothbrushing? (7) Do you brush your teeth after lunch regularly? (8) Do you brush your teeth before bedtime regularly? Each question concerning self‐efficacy in oral hygiene was scored using a 4‐point scale, ranging from 1 (strongly disagree) to 4 (strongly agree). Overall, the maximum score was 23 points, with a higher score indicating better oral health practice and self‐efficacy related to oral hygiene.

### 2.3 Oral Health Impact Profile‐14 (OHIP‐14)

The OHIP‐14 (Slade, 1997) consists of 14 questions related to oral quality of health. The OHIP‐14 covers seven domains: functional limitation, physical pain, psychological discomfort, physical disability, psychological disability, social disability, and handicap, allowing the oral quality of life estimation over the past month. The total score ranged from 0 to 56 points. Items were scored on a 5‐point scale, with 0: Never, 1: Rarely, 2: Sometimes, 3: Often, 4: Very often. A lower score indicated better oral quality of life. The OHIP‐14 has been used frequently (Tuk et al., 2021), and its validity in Japan has been demonstrated (Ikebe et al., 2004).

### 2.4 Development of “Toothbrushing Timer with Information on Toothbrushes” app

The “Toothbrushing Timer with Information on Toothbrushes” app was created using Microsoft Power Apps (Microsoft Inc.) and Excel (Microsoft Inc.) and could be accessed from a smartphone, tablet, and PC through Microsoft 365 (Microsoft Inc.) with the Hiroshima University license. The main screen of our app consisted of the “start search” button, a “toothbrushing timer,” and “calendar.” The “toothbrushing timer” was set to 3 min because toothbrushing for 3 min was recommended based on previous studies (Ganss et al., 2009; Hayasaki et al., 2014; Kaneyasu et al., 2020; Madlena et al., 2004). In contrast to a mobile phone or kitchen timer, the “toothbrushing timer” did not require the participant to set the time for 3 min. Participants only needed to push the “toothbrushing timer” button and automatically measured toothbrushing duration for 3 min while they were searching for the next new toothbrush within our app. Therefore, the participants were suggested to use the “toothbrushing timer” when they were brushing their teeth. The calendar application was furnished supplementally to help replace the next toothbrush at the appropriate time. The search screen was connected to the “start search” button on the main screen. Our application included data on 548 kinds of toothbrushes except electronic toothbrushes sold in Japan, and participants were able to learn characteristics of toothbrushes they used. Additionally, participants were able to find suitable toothbrushes for the user by entering the search word concerning the desired use of the toothbrush, the users' oral condition (e.g., gingivitis, periodontitis, caries, and orthodontics), and the user's development stage (e.g., adult, child, and mixed dentition period) into the search box. The search results provided information in terms of 13 items (Table 1), for example, “Product name,” “Subject/uses,” “Bristle materials,” and “Beneficial characteristics of toothbrush.” The performance test of our app was confirmed individually by researchers after performing the preview function on the creation screen.

**Table 1:** Items included in the Toothbrushing Timer with Information on Toothbrushes application.

No.	Item
1	Manufacturer/distributor
2	Product name
3	Subject/uses
4	Bristles stiffness
5	Prices
6	Bristles materials
7	Characteristics of bristles
8	Length of bristles
9	Diameter of bristles
10	Beneficial characteristics of toothbrush
11	Heat‐resistance of temperature
12	Color variation
13	Materials of handle

### 2.5 Evaluation of the “Toothbrushing Timer with Information on Toothbrushes” App

To examine the utility of our app, the participants answered the questionnaire items at T1 using the QR code as follows: “Was this app easy for you?” and “Would you like to recommend this app to others?” The responses to both questions were “yes” or “no.”

### 2.6 Statistical analysis

The Mann–Whitney *U* test was used to evaluate significant differences in age, and between the health professional and non‐health professional groups at both T0 and T1. Fisher's exact test was used to evaluate significant differences in the characteristics of participants between the two groups. The Wilcoxon signed‐rank test was used to evaluate significant changes between T0 and T1 scores for both the health professional and non‐health professional groups. Statistical analysis was performed using JMP Pro software (version 15.0.0, SAS Institute Inc.). The level of significance was set at *p* < .05.

## 3 RESULTS

### 3.1 Participants of this study

**Figure 1:**
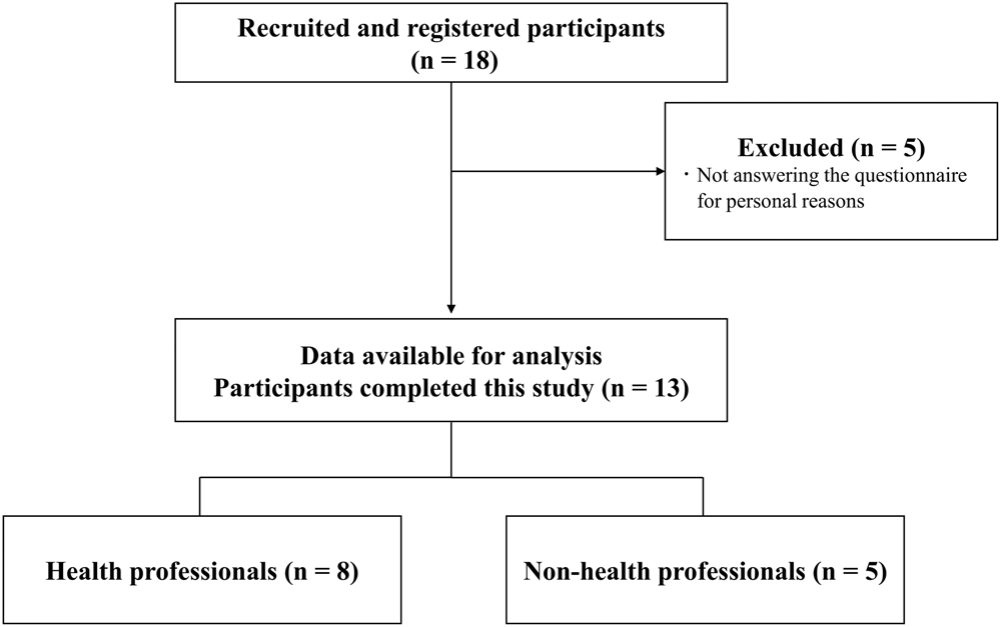
Flow diagram showing the participant selection criteria for the study.

A total of 18 participants were initially registered for the study, of whom five were excluded from the analysis as they did not answer the digital questionnaire for personal reasons. Finally, 13 participants (mean age ± standard deviation: 34.46 ± 14.74 years) completed the survey with a follow‐up of 1 month (Figure 1). Table 2 summarizes the characteristics of participants. The 13 participants were grouped into health professionals (*n* = 8) and non‐health professionals (*n* = 5), based on the occupation indicated in the questionnaire (Table 3, Figure 1). None of the participants reported having caries or undergoing periodontal treatment; therefore, they were considered to have a relatively good oral condition.

**Table 2:** Characteristics of participants.

Variables	
Age (mean ± SD) (years)	34.46 ± 14.74
Sex (*n*, %)	
Male	0 (0)
Female	13 (100)
Degree and position (*n*, %)	
Students	6 (46.15)
Staffs	7 (53.85)
Occupation (*n*, %)	
Health professionals	8 (61.54)
Non‐health professionals	5 (38.46)
Under caries and periodontal treatment (*n*, %)	
Yes	0 (0)
No	13 (100)
Under orthodontic treatment (*n*, %)	
Yes	3 (23.08)
No	10 (76.92)
Undergo regular dental checkups (*n*, %)	
Yes	8 (61.54)
No	5 (38.46)

Abbreviation: SD, standard deviation.

**Table 3:** Comparison between the health professional and non‐health professional groups.

Variables	Health professionals (*n* = 8)	Non‐health professionals (*n* = 5)	*p* Value
Age (mean ± SD) (years)	29.0 ± 15.85	43.2 ± 7.56	.04^a^
Under orthodontic treatment (*n*, %)			
Yes	3 (37.5)	0 (0)	.23^b^
No	5 (62.5)	5 (100)	
Undergo regular dental checkups (*n*, %)			
Yes	4 (50)	4 (80)	.56^b^
No	4 (50)	1 (20)	

Abbreviation: SD, standard deviation.

^a^ Mann–Whitney *U* test.

^b^ Fisher's exact test. *p* < .05 were considered statistically significant.

### 3.2 Oral health practice and self‐efficacy related to oral hygiene

There were no significant differences in oral health practice and self‐efficacy related to oral hygiene scores between T0 and T1 within the health professional and non‐health professional groups. Additionally, there were no significant differences between the health professional and non‐health professional groups at T0 and T1. However, both the health professional and non‐health professional groups had increased toothbrushing time scores at T1 (health professionals: 2.88 ± 0.35, non‐health professionals: 3.00 ± 0.00) than at T0 (health professionals: 2.75 ± 0.46, non‐health professionals: 2.60 ± 0.55; Table 4). Furthermore, both groups had higher scores at T1 (health professionals: 3.00 ± 0.54, non‐health professionals: 2.40 ± 0.55) than at T0 (health professionals: 2.75 ± 0.46, non‐health professionals: 2.00 ± 0.71; Table 4) on the item of “Do you think you keep your teeth clean by toothbrushing?” and the total scores of both oral health practice and self‐efficacy related to oral hygiene were increased at T1 compared to T0.

**Table 4:** Changes in the oral health practice and self‐efficacy related to oral hygiene.

	Health professionals			Non‐health professionals		
	T0 (*n* = 8)	T1 (*n* = 8)	*p* Value^a^	T0 (*n* = 5)	T1 (*n* = 5)	*p* Value^a^
	Mean ± SD	Mean ± SD		Mean ± SD	Mean ± SD	
Oral health practice						
Frequency of toothbrushing (per/day)	2.88 ± 0.35	2.88 ± 0.35	>.99	2.40 ± 0.55	2.60 ± 0.55	>.99
Toothbrushing time (min)	2.75 ± 0.46	2.88 ± 0.35	>.99	2.60 ± 0.55	3.00 ± 0.00	.05
Using cleaning aids (dental floss or interdental brushes)	1.0 ± 0.00	1.00 ± 0.00	>.99	1.00 ± 0.00	1.00 ± 0.00	>.99
Using the oral rinse	0.25 ± 0.46	0.38 ± 0.52	>.99	0.20 ± 0.45	0.20 ± 0.45	>.99
Frequency of replace the toothbrush	2.50 ± 0.53	2.50 ± 0.54	>.99	2.00 ± 0.00	2.00 ± 0.00	>.99
Total oral health practice	9.38 ± 1.06	9.63 ± 1.19	.75	8.20 ± 1.00	8.80 ± 0.84	.25
Self‐efficacy related to oral hygiene						
Do you think you keep your teeth clean by toothbrushing?	2.75 ± 0.46	3.00 ± 0.54	.50	2.00 ± 0.71	2.40 ± 0.55	.50
Do you brush your teeth after lunch regularly?	3.13 ± 0.64	3.13 ± 0.64	>.99	2.60 ± 0.89	2.60 ± 0.55	.99
Do you brush your teeth before bedtime regularly?	3.50 ± 0.76	3.75 ± 0.46	.50	3.80 ± 0.45	3.60 ± 0.55	.99
Total self‐efficacy related to oral health	9.38 ± 1.60	9.88 ± 1.46	.31	8.40 ± 1.95	8.60 ± 1.14	.99
Total oral health practice and self‐efficacy related to oral hygiene	18.75 ± 1.83	19.50 ± 2.33	.22	16.60 ± 2.20	17.40 ± 1.52	.38

*Note*: 1.0 indicates “yes” and 0 indicates “no” in using cleaning aids and using the oral rinse. Frequency of replacing the toothbrush was scored as 1 point in cases where the participant replaced the toothbrush at 6 months or more after use of toothbrush, scored as 2 points in cases where the participant replaced the toothbrush at more than 3 months and less than 6 months after use or when the bristles were splayed, and scored as 3 points in cases where the participant replaced the toothbrush within 1 month after use.

Abbreviations: SD, standard deviation; T0, baseline; T1, 1 month.

^a^ Wilcoxon signed‐rank test. Values of *p* < .05 were considered statistically significant.

### 3.3 OHIP‐14 scores

There were no significant differences in the OHIP‐14 scores between T0 and T1 within the health professional and non‐health professional groups. Furthermore, there were no significant differences between groups at T0 and T1. However, both groups had lower OHIP‐14 total scores at T1 (health professionals: 8.88 ± 5.52, non‐health professionals: 6.80 ± 7.12) than at T0 (health professionals: 9.25 ± 5.04, non‐health professionals: 12.60 ± 5.51; Table 5).

**Table 5:** Changes in the OHIP‐14 scores.

	Health professionals			Non‐health professionals		
	T0 (*n* = 8)	T1 (*n* = 8)	*p* Value^a^	T0 (*n* = 5)	T1 (*n* = 5)	*p* Value^a^
	Mean ± SD	Mean ± SD		Mean ± SD	Mean ± SD	
Functional limitation (1, 2)	1.13 ± 0.99	1.13 ± 0.99	>.99	2.00 ± 1.23	0.80 ± 0.84	.25
Physical pain (3, 4)	1.63 ± 1.30	1.38 ± 1.06	.75	2.40 ± 1.52	1.60 ± 1.52	>.99
Psychological discomfort (5, 6)	1.88 ± 0.83	1.50 ± 0.76	.25	2.20 ± 0.45	1.00 ± 1.00	.13
Physical disability (7, 8)	1.00 ± 0.93	1.00 ± 0.93	>.99	1.40 ± 0.90	0.80 ± 1.30	.63
Psychological disability (9, 10)	1.75 ± 1.17	2.00 ± 1.20	.75	2.20 ± 0.84	1.20 ± 1.30	.25
Social disability (11, 12)	0.63 ± 0.92	1.00 ± 0.93	.50	1.20 ± 0.84	0.60 ± 0.89	.50
Handicap (13, 14)	1.25 ± 0.89	0.88 ± 0.84	.50	1.20 ± 0.84	0.80 ± 1.10	.50
Total OHIP‐14	9.25 ± 5.04	8.88 ± 5.52	.69	12.60 ± 5.51	6.80 ± 7.12	.25

Abbreviations: OHIP‐14, Oral Health Impact Profile‐14; SD, standard deviation; T0, baseline; T1, 1 month.

^a^ Wilcoxon signed‐rank test. Values of *p* < .05 were considered statistically significant.

### 3.4 Evaluation of our app

More than 84% (11/13) of the participants answered that our app was easy to use, and more than 61% (8/13) responded that they would recommend it to others.

## 4 DISCUSSION

The results of this study did not show significant changes in oral health awareness and practice between the groups. However, this study revealed some possible trends (improvement of oral health awareness and oral health practice after using our app) that should be validated in future investigations. Recently, numerous mobile applications have become available (Underwood et al., 2015). To the best of our knowledge, few studies have focused on apps that aid in learning about toothbrush characteristics independent of the manufacturer or distributor. Our app is able to connect with any digital device, such as PCs, tablets, and smartphones, using Microsoft 365 (Microsoft Inc.). Furthermore, our app is equipped with a “toothbrushing timer” to facilitate the establishment of healthy oral hygiene practices. Thus, we consider that our application may contribute to the awareness and practice of good oral hygiene. If people want to find toothbrushes by searching on the Internet, they are often confused because the information provided is not clearly arranged in list form. On the contrary, our app is easy to use and offers information of many types of toothbrushes when replacing the toothbrush. Therefore, our application may contribute to the maintenance of good oral health by regular toothbrushing with an appropriate toothbrush.

To date, several reports have used a questionnaire regarding oral health practice and self‐efficacy in oral hygiene to evaluate the efficacy of oral health education (Bonabi et al., 2019; Haque et al., 2016; Kumar et al., 2022; Wei et al., 2021). The results of previous studies showed that the oral health practice scores of an intervention group who received oral health education increased, in line with our results (Bonabi et al., 2019; Haque et al., 2016). In this study, the participants showed an increase in tooth‐brushing time and in tendency of self‐efficiency toward thorough toothbrushing cleanly after the intervention at T1 compared to T0, and similar results were observed by Haque et al. (2016) and Wei et al. (2021). Particularly, non‐health professionals exhibited greater improvement in oral health practice in comparison to health professionals.

The scores of the OHIP‐14 in this study were lower than those of patients with spinal cord injury (Pakpour et al., 2016) or those undergoing periapical surgery (Tuk et al., 2021), because the participants recruited in the present study were healthy volunteers. Moreover, non‐health professionals showed a larger decrease in these scores than health professionals after 1 month. In contrast, differences in OHIP‐14 scores among those in the health professional group changed only slightly. Thus, non‐health professionals were more affected by oral hygiene interventions and, therefore, our app may have had a stronger influence on the non‐health professional group than on the health professional group. Additionally, both the health professional and non‐health professional groups tended to show a decrease from T0 to T1 in the total OHIP‐14 score. Hence, our app can be beneficial for all users, especially those who do not work in a health‐related occupation, for the maintenance and improvement of oral health‐related quality of life. Moreover, the quality of life related to oral health, which was examined using the OHIP‐14, was significantly associated with oral hygiene practice, such as toothbrushing habits (Avasthi et al., 2022; Sinha et al., 2020). Therefore, the quality of life related to oral health may have affected the oral health practices of participants.

We used a 1‐month follow‐up period, following previous studies that used a 1‐month follow‐up period (Van Leeuwen et al., 2019; Shida et al., 2020). There are also other studies that used different follow‐up periods to assess the efficacy of a mobile app, for example, 2 weeks (Humm et al., 2020), 1 week (Jacobson et al., 2019), and 6 weeks (Ki et al., 2021). Long‐term intervention tends to result in users becoming bored with using digital devices (Shida et al., 2020), such as e‐learning materials and mobile apps. Therefore, the 1‐month period was considered ideal because it was neither too long nor too short.

Our app includes both a toothbrushing timer and information concerning the toothbrushes. To our knowledge, this is the first report of an app including both a toothbrushing timer and toothbrush information. We collected the data (i.e., people applicable, the size of toothbrush, characteristics of toothbrush materials, and beneficial characteristics of the toothbrush) of more than 500 kinds of toothbrush released from Japanese companies using the latest user's manual for each toothbrush. Therefore, our app enables many people to learn characteristics of toothbrushes and find a suitable toothbrush as quickly as possible. Additionally, our app includes general information on recommended characteristics for different patients (i.e., patients with periodontitis, orthodontic patients, and patients with dental cavity). Therefore, using this app, such patients may easily find the best toothbrushes suitable for their oral health conditions by entering the search word oral condition. Furthermore, people may learn the characteristics of toothbrush they are currently using. Our app may help healthy people and those with oral health issues find and use the appropriate toothbrush. Our app may improve oral health condition by establishing regular toothbrushing behavior with a proper toothbrush.

Our “Toothbrushing Timer with Information on Toothbrushes” app tended to maintain and improve oral health awareness and practice for users.

There were some limitations in this study. First, the impact of our app on objective oral health indices, such as gingivitis and dental plaque, is unknown. Therefore, we could not sufficiently clarify the usefulness of our app. Data on oral hygiene status as well as oral health awareness and practice in participants is necessary to calculate the correct sample size in the next study. It is also unknown whether the participants followed the recommendation made by the app and replaced their toothbrushes within a month. Hence, it is necessary to conduct further studies with an extended study period to reveal our app's impact on the gingival tissue and dental plaque accumulation and develop a reminder function in our app for informing the time point for substituting the toothbrush. Second, there was a significant difference in age at baseline (T0) between the groups, all participants were female, and the sample size was very small. In addition, we could not include participants who did not use the app as a control group in this study. This can be attributed to COVID‐19 which affected the recruiting participants of our research; there were limited participants at the university that could be recruited voluntarily. Therefore, it is necessary to eliminate bias as much as possible by increasing the number of participants and conducting a randomized controlled trial. Furthermore, it is important to compare the hygiene levels of participants with and without use of the app. Third, our app did not include information concerning electric toothbrushes as it was considered that manual toothbrushes are the most widely used instruments to control tooth surface and gingival tissue health (Ranzan et al., 2019). However, electric toothbrushes are also used widely (Deery et al., 2004; Wang et al., 2020). Therefore, it is necessary to develop a new app including information about electric toothbrushes, and to verify the effect of the app on oral health awareness and practice. Finally, according to free responses to descriptive questions on the digital questionnaire, the participants suggested that our app should add a photograph of each toothbrush and provide appropriate toothbrushing educational materials. Hence, we intend to improve the app by addressing these issues in the future.

## 5 CONCLUSION

Our “Toothbrushing Timer with Information on Toothbrushes” app tended to maintain and improve oral health awareness and practice for users. However, this pilot study failed to show the efficacy of the app owing to small sample size. Therefore, it is necessary to evaluate the impact of our app on oral health conditions and clarify its significance in a future study with a larger sample size.

## AUTHOR CONTRIBUTIONS

**Yoshino Kaneyasu**: Conceptualization; data curation; investigation; methodology; software; writing—original draft; writing—review and editing. **Hideo Shigeishi**: Formal analysis; software; writing—review and editing. **Masaru Sugiyama**: Supervision; writing—review and editing. **Kouji Ohta**: Funding acquisition; project administration; software; supervision; writing—review and editing. All authors have read and agreed to the published version of the manuscript.

## CONFLICT OF INTEREST STATEMENT

The authors declare no conflict of interest.

## ETHICS STATEMENT

This study design was approved by the Ethics Committee of Hiroshima University (E‐2766). Informed consent was obtained from all subjects involved in the study.

## Data Availability

All relevant data are included in this article.
